# Prognosis patients with COVID-19 using deep learning

**DOI:** 10.1186/s12911-022-01820-x

**Published:** 2022-03-26

**Authors:** José Luis Guadiana-Alvarez, Fida Hussain, Ruben Morales-Menendez, Etna Rojas-Flores, Arturo García-Zendejas, Carlos A. Escobar, Ricardo A. Ramírez-Mendoza, Jianhong Wang

**Affiliations:** 1 Escuela de Ingeniería y Ciencias, Tecnologico de Monterrey, Av. Eugenio Garza Sada 2501 Sur, Tecnológico, 64849 Monterrey, N.L. Mexico; 2 General Motors, Pontiac, MI USA; 3 School of Electronic Engineering and Automation, Jiangxi University of Science and Technology, Ganzhou, China

**Keywords:** Deep learning, Random forest, Coronavirus, COVID-19, Prognosis, Mortality risk prediction

## Abstract

**Background:**

The coronavirus (COVID-19) is a novel pandemic and recently we do not have enough knowledge about the virus behaviour and key performance indicators (KPIs) to assess the mortality risk forecast. However, using a lot of complex and expensive biomarkers could be impossible for many low budget hospitals. Timely identification of the risk of mortality of COVID-19 patients (RMCPs) is essential to improve hospitals' management systems and resource allocation standards.

**Methods:**

For the mortality risk prediction, this research work proposes a COVID-19 mortality risk calculator based on a deep learning (DL) model and based on a dataset provided by the HM Hospitals Madrid, Spain. A pre-processing strategy for unbalanced classes and feature selection is proposed. To evaluate the proposed methods, an over-sampling Synthetic Minority TEchnique (SMOTE) and data imputation approaches are introduced which is based on the K-nearest neighbour.

**Results:**

A total of 1,503 seriously ill COVID-19 patients having a median age of 70 years old are comprised in the research work, with 927 (61.7%) males and 576 (38.3%) females. A total of 48 features are considered to evaluate the proposed method, and the following results are achieved. It includes the following values i.e., area under the curve (AUC) 0.93, F2 score 0.93, recall 1.00, accuracy, 0.95, precision 0.91, specificity 0.9279 and maximum probability of correct decision (MPCD) 0.93.

**Conclusion:**

The results show that the proposed method is significantly best for the mortality risk prediction of patients with COVID-19 infection. The MPCD score shows that the proposed DL outperforms on every dataset when evaluating even with an over-sampling technique. The benefits of the data imputation algorithm for unavailable biomarker data are also evaluated. Based on the results, the proposed scheme could be an appropriate tool for critically ill Covid-19 patients to assess the risk of mortality and prognosis.

## Background

Coronavirus is a large family of viruses that can cause common colds, severe acute respiratory syndrome (SARS), and Middle East respiratory syndrome (MERS). It has been proven that the root cause is a new type of virus called the 2019 Novel Coronavirus (COVID-19). The World Health Organization (WHO) and the Centres for Disease Control and Prevention (CDC) are closely monitoring the development of the virus and how to prevent and treat diseases caused by the COVID-19. The COVID-19 epidemic has caused an astonishing loss of life around the world and poses an exceptional challenge to public health and the food system. The social and economic interruption triggered by this epidemic is damaging; millions of people are at risk of falling into extreme hardship, while millions of businesses face an existential threat. Approximately half of the world’s 3.3 billion global personnel are at risk of losing their incomes [1–17].

## Related works

Recently many algorithms have been developed to diagnose the COVID-19 outbreak [18–22]. A predictive model of COVID-19 disease progression was proposed by [23] using multivariate analysis (Cox proportional regression). Pourhomayoun et al. [24] proposed an ML algorithm to accurately predict the mortality risk of COVID-19 patients. In [25], a Gaussian process regression (GPR) model with optimized hyperparameters was used to predict the mortality rate using five different countries (Turkey, Spain, Sweden, France and Pakistan) datasets [26]. An extreme gradient boosting (XGBoost) classifier was proposed to model the probability of requiring mechanical ventilation within the next 24 h, using data from the first 2 h after admission [27]. In [28], attempted to predict the occurrence of major adverse cardiac events (MACE) in acute myocardial infarction (AMI) patients, during the 1, 6 and 12 months follow up periods after hospital admission using ANN. A mortality risk calculator was established based on the XGBoost model, and the patients’ dataset was collected from hospitals in Spain (HM Hospitals) and Italy (ASST Cremona) [29]. A deep neural network transfer model based on a convolutional neural network (CNN) was proposed to diagnose a patient with COVID-19 by analysing their lungs’ X-ray images [30]. In [31], proposed a CNN transfer learning model to diagnose COVID-19 patients using X-ray and CT-Scan images and the gradient weighted class activation mapping (GRAD-CAM) technique [32, 33]. Yadaw et al. [34] developed a mortality prediction model using an XGBoost algorithm. In [35], a multivariate regression model was introduced based on clinical characteristics to predict ICU admissions and mortality in COVID-19 patients. Wearable technologies have been developed to identify patients with COVID-19 [13, 20, 36].

Besides, most recent studies have been reported to understand and diagnose the patients with COVID-19 [37–42] such as temporal deep learning [43], data-driven based extreme gradient boosting (XGBoost) [44], deep learning with regression analysis [45], biomarkers-based [46], machine learning and clinical data based [35, 47, 48], statistical neural network (NN) and DL [49–51], boosted random forest [52], CNN-LSTM, CNN-RNN and CNN-ML based on X-ray images [53–57].

Early identification of COVID-19 patients is crucial for the severity of the risk. The patients with high risk are to be identified earlier than those with very low risk for this critical disease. Moreover, not every hospital has the resources, budget, time, staff, equipment, etc., to conduct many complicated tests before needing to decide the risk. A mortality risk calculator for COVID-19 is designed to be as accurate as possible and uses a minimum number of features to produce an acceptable prediction rate. The proposed methodology will help to easily prognose the patient’s survival rate.

Though there are already a lot of Machine Learning (ML) algorithms that have been proposed for the prediction of patients with COVID-19, most of them have not reached optimal results, because of the lack of useful data, or because they are highly biased to only a certain population.

## Study contributions

The main contributions of this research work are as follow:

1. A total of 48 vital features are considered including biomarkers to predict the mortality risk of COVID-19 patients and the trade-off between performance feature and sample space.
2. The data imputation scheme is introduced which is based on the K-Nearest Neighbor and over-sampling Synthetic Minority TEchnique (SMOTE) approaches.
3. To develop the proposed DL model, a web application of Amazon Web Services (AWS) has been used and is intended to help frontline physicians in clinical decision making under time-sensitive and resource-constrained conditions for COVID-19 patients.
4. The prediction performance of DL models is investigated using basic features against specialized features.
5. The oversample and data augmentation techniques are introduced to check the effect of the DL model.
6. The results of the proposed DL model are compared against a random forest (RF), support vector machine (SVM), artificial neural network (ANN), XGBoost, logistic regression (LR) models to assess its benefits when attempting to reduce the false-negative rate (FNR).
7. Further, the benefits of using over-sampling and data imputation techniques (i.e., SMOTE and KNN imputation) are reported.
8. Based on the MPCD score, the proposed DL outperforms on every dataset including an over-sampling technique.

Organization of the paper: “Datasets and Pre-process” section contains materials and methods, including datasets and pre-processing of the datasets. "Results and discussion" comprises the results and discussion, including comparative analysis and advantages and disadvantages of the research work. “Conclusion and future work” section covers the conclusion and future work of the research.

## Datasets and pre-process

### Database description

The datasets of the patients with COVID-19 have been collected from HM Hospitals Madrid, Spain. It contains the anonymized records of 2,307 patients with COVID-19. The database was divided into six different sections, each section contained a different type of data of each patient. The common key among every file is the patient ID feature, which helps to identify patients across every section of the database. The summary of the database is shown in Table 1.

**Table 1:** Complete raw database description

Section	Description
1	Demographic data: patient ID, age, gender, diagnosis
2	(positive/negative/pending), admission/discharge date, SpO2, temperature, heart rate, blood pressure, etc
3	Prescribed medication: daily dose and duration
4	Evolution of vital signs: SpO2, heart rate, temperature, blood pressure, and blood glucose values
5	Laboratory tests with date, results, and units
6	comorbidities are coded based on [World Health Organization (WHO)]

### Data cleansing

**Fig. 1:**
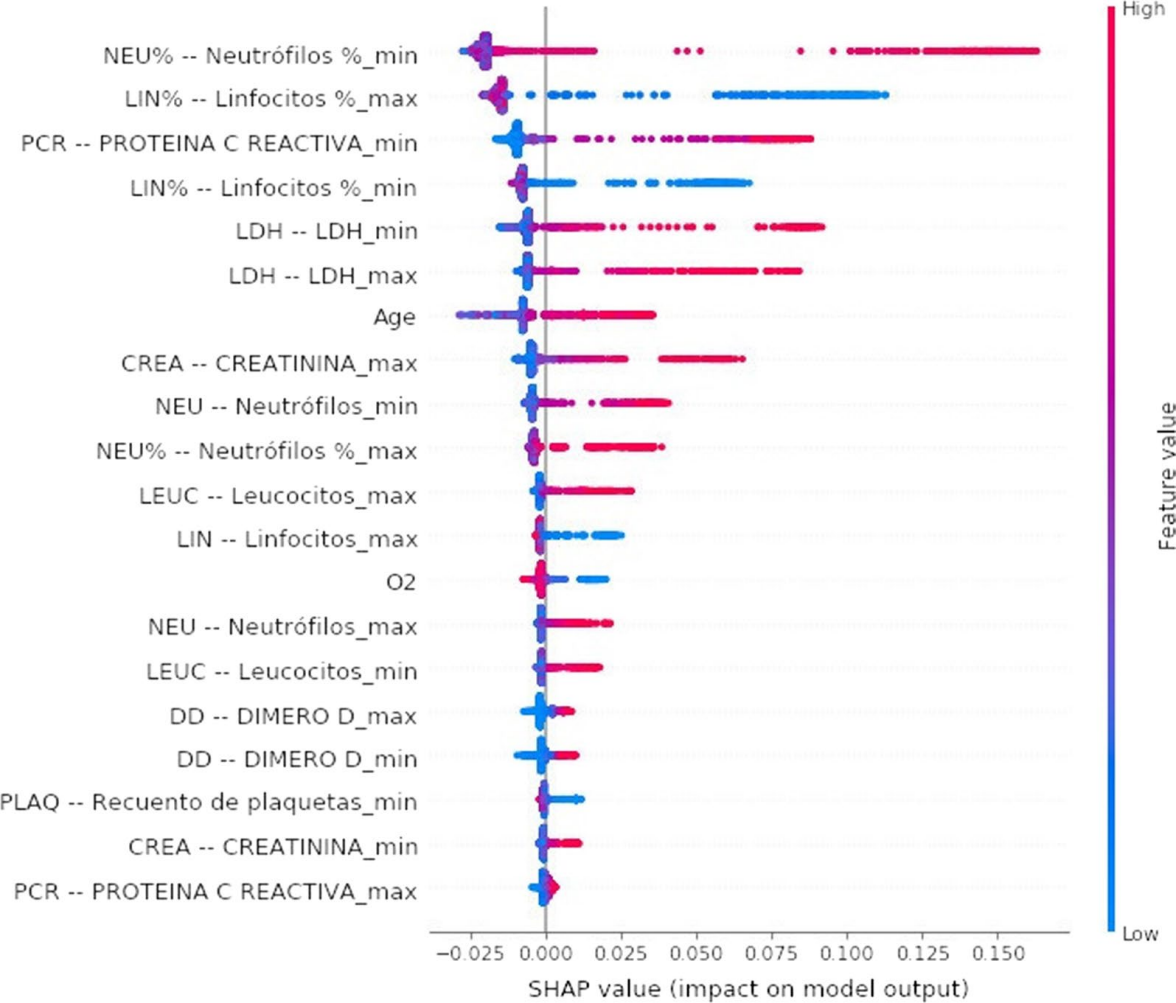
SHAP values of the selected features accordingly (dataset 8)

This raw database had five main technical challenges: (1) Incomplete record, (2) Different units, (3) Combination of categorical and numerical values. (4) Irrelevant and redundant, (5) Unbalanced classes. The database from all 2307 available records was filtered using the following standards: let alone patients with a COVID-19 positive diagnosis, discharged or confirmed to die, different from 0 years of age, their registered value of SpO2. After applying all these filters, only 1503 patients were left. The RF algorithm has been used to select the features with the highest predictive power, and to decrease the feature space by analysing the importance assigned to each feature by the algorithm. For this purpose, the SHapley Additive exPlanations (SHAP) values [58] was used to estimate the impact/weight of each input variable in the prediction. The SHAP value graph is a graphical visualization of how much a feature contributes to the model’s prediction. A large positive SHAP value indicates the feature is very relevant to detect positive outputs, while a large negative value is associated with negative output. The colour bar shows the feature value associated with the given SHAP value, while the thickness of a feature’s line indicates the number of samples present in the dataset for the given feature value, and the SHAP values are shown in Fig. 1.

In this research work, some biomarkers were selected including prothrombin activity, creatinine, D-dimer, ferritin, immunoglobulin-G, immunoglobulin-M, interleukin-6, lactate, LDH, leukocytes (count and %), lymphocytes (count and %), neutrophils (count and %), C-reactive protein, platelets, prothrombin time, and troponin. Since the sampling frequency of lab tests is inconsistent, simple time series statistical representation, such as maximum and minimum values, was chosen to represent these characteristics of the biomarkers. Therefore, two more features were added for every biomarker, one for the maximum and another one for the minimum values. Features 4 through 10 are categorical data in [True; False], while every other feature value is considered as numerical data in real (R). All available features are presented in Table 2.

**Table 2:** Index, acronym, and name of the features

S. No	Acronym	Feature Name	S. No	Acronym	Feature Name
1		Patient ID	25	IgM (min)	IgM (Immunoglobulin M) (min)
2		Age	26	IgG (min)	IgG (Immunoglobulin G) (min)
3	SpO2	SpO2 Oxygen saturation (O2)	27	TNI (min)	Troponin (min)
4		Gender,	28	PA (min)	Prothrombin activity (min)
5		Discharge motive (Label)	29	PT (min)	Prothrombin time (min)
6		kidney failure (N17)	30	LIN (% max)	Lymphocytes (% max)
7		hypertension (I10)	31	LIN (max)	Lymphocytes (max)
8		diabetes (E11)	32	LEUC (max)	Leukocytes (max)
9		heart disease (I25)	33	NEU (max)	Neutrophils (max)
10		respiratory distress (J80)	34	NEU (% max)	Neutrophils (% max)
11	LIN (% min)	Lymphocyte (% min)	35	PLAQ (max)	Platelet count (max)
12	LIN (min)	Lymphocyte (min)	36	PCR (max)	C-reactive protein (max)
13	LEUC (min)	Leukocyte (min)	37	DD (max)	Dimer D Max
14	NEU (min)	Neutrophils (min)	38	CREA (max)	Creatinine (max)
15	NEU (% min)	Neutrophils (% min)	39	LDH (max)	LDH (max)
16	PLAQ (min)	Platelet count (min)	40	IL6 (max)	Interleukin 6 (max)
17	PCR (min)	C-reactive protein (min)	41	LEULCR (max)	Leukocyte’s count (max)
18	DD (min)	D Dimer (min)	42	LAC (max)	Lactate (max)
19	CREA (min)	Creatinine (min)	43	FER (max)	Ferritin (max)
20	LDH (min)	LDH (min)	44	IgM (max)	IgM (Immunoglobulin M) (max)
21	IL6 (min)	Interleukin 6 (min)	45	IgG (min)	IgG (Immunoglobulin G) (max)
22	LEULCR (min)	Leukocyte’s count (min)	46	TNI (max)	Troponin (max)
23	LAC (min)	Lactate (min)	47	PA (max)	Prothrombin activity (max)
24	FER (min)	Ferritin (min)	48	PT (max)	Prothrombin time (max)

### Data distribution

**Fig. 2:**
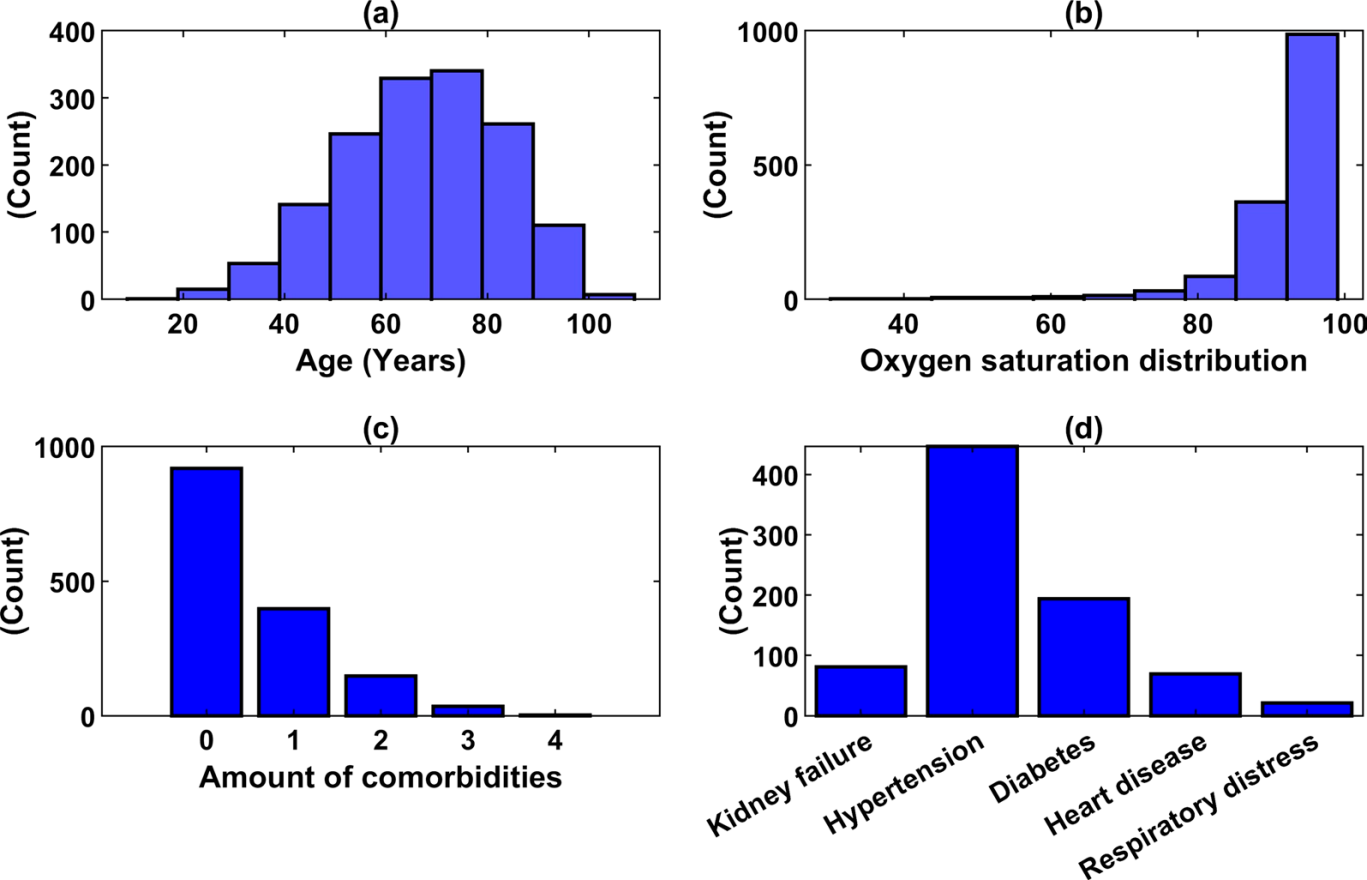
Distribution of age (**a**), oxygen saturation (SpO2) (**b**), multiple comorbidities (**c**) and comorbidities (**d**)

In Fig. 2, it can be seen that the average age distribution of patients after the normal distribution is about 70 years old. Oxygen saturation values have a mean of 92.28, with a couple of lower outliers, which suggests a more severe disease state according to literature. A clear unbalance of the classes is observed, with only 16.5% of deceased patients. As for comorbidities, there are 919 patients with none of the selected comorbidities, 398 patients with only 1 comorbidity, 148 patients with 2 of them, 35 patients with 3, 3 patients with 4 comorbidities, and no patients with every comorbidity. The most common comorbidity among patients is hypertension. Since the original dataset contains a lot of missing cells, the sample size reduces as the number of features increases. Tables 3 and 4 show the filtered database distribution and biomarkers of the patients (1,503) with COVID-19, respectively.

**Table 3:** Filtered database distribution according to key features

Feature	Detail	Number of patients (%)
Gender	Male	927 (61.7%)
Comorbidities	Kidney failure	81 (5.4%)
	Hypertension	446 (29.7%)
	Diabetes	194 (12.9%)
	Heart disease	69 (4.6%)
	Respiratory distress	21 (1.4%)
Discharge motive	Deceased	248 (16.5%)

**Table 4:** The minimum and maximum range of biomarkers

Name of biomarker	Mean value min–max
LEUC (× 103/µL)	6.03–9.85
LIN (× 103/µL)	1.00–1.65
LIN%	13.90–25.03
NEU (× 103/µL)	4.10–7.84
NEU%	63.23–78.66
PLAQ (× 103/µL)	206.42–320.81
Cr (mg/dL)	0.84–1.09
PCR (mg/L)	38.84–130.69
LDH (U/L)	482.91–726.57
DD (ng/mL)	1194.71–4509.60
IL6 (pg/mL)	192.74–239.50
LAC (mmol/L)	1.73–2.29
FER (ng/mL)	1150.13–1526.91
TNI (ng/L)	27.01–36.47
PA (%)	70.58–80.93
PT (s)	13.70–16.55

### Pre-processing dataset

**Fig. 3:**
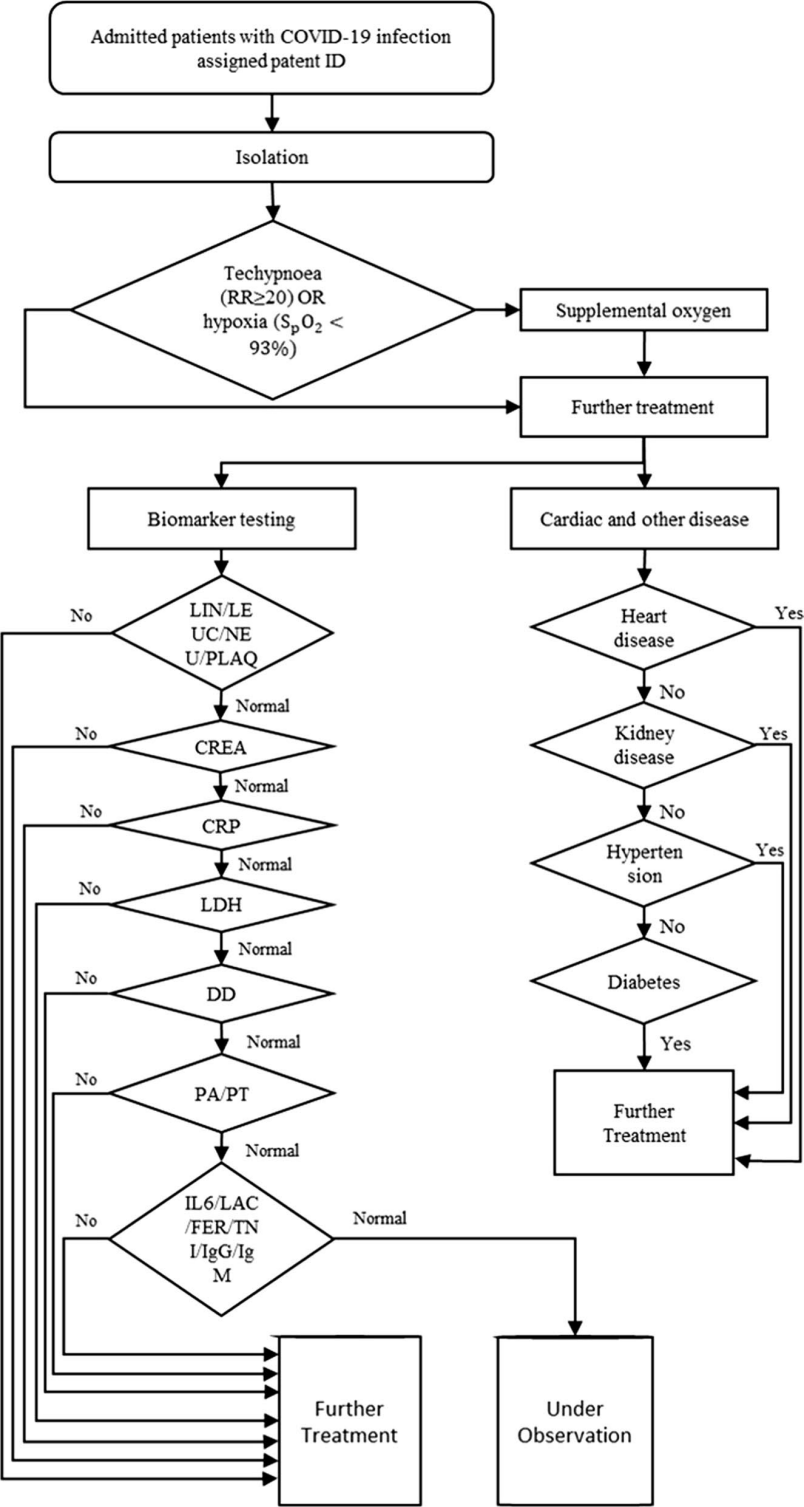
The overall proposed feature selection scheme

To increase the training data availability, a pre-processing algorithm [59] was employed, it is a Greedy-like algorithm that at each iteration maximizes the number of samples by selecting the column (feature) with more rows (samples) available. Since the original dataset contains a lot of missing cells, the sample size reduces as the number of features increases. The eight sub-datasets pose a trade-off between the number of features and the number of samples, as the subset cannot be predetermined with highly distinguished information, the learning algorithm was applied to all of them. Since normalizing data generally accelerates learning rate and leads to faster convergence [60] the remaining numeric features have been re-scaled using the min–max normalization method [61]. By examining the datasets, it was created into eight sub-datasets with different features. The features and sub-datasets are illustrated in Fig. 3 and Table 5.

**Table 5:** The number of features in every defined sub-dataset

Data subsets	No. of features	No. of samples
1	10	1503
2	22	1449
3	24	1434
4	26	1428
5	28	1419
6	30	1341
7	34	1291
8	48	683

### Methodologies

**Fig. 4:**
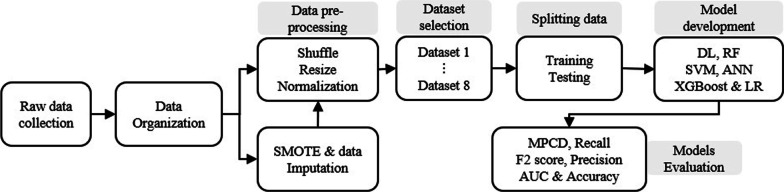
The overall proposed architecture for the risk detection of patients with COVID-19 infection

The mortality risk calculator for the COVID-19 patients has multiple steps; (1) Collection of raw data, (2) Data pre-processing, (3) Over-sampling & data imputation and splitting the data, (4) Model developments and (5) Model evaluation. The proposed overall procedure is shown in Fig. 4.

### Deep learning (DL)

The basic idea behind DL is to stack many shallow multi-layer algorithms to obtain a more abstract representation of features as the network gets deeper [62]. DL has recently gained popularity, particularly in the field of computer vision but is rapidly moving towards different areas, such as diagnosis and prognosis in the medical field [63]. The quintessential DL algorithm is the ANN. ANNs are a type of ML algorithm roughly based on the biological neurons of the brain and the way that they are interconnected with one another to learn complex abstract representations. In this research work, the DL model with binary cross-entropy as the loss function and the Adam algorithm as an optimizer to adjust the network’s weights have been used. The model has 3 hidden layers with 17, 10 and 5 neurons, respectively. The mini-batch optimization technique was utilized. For the binary classification problem, the sigmoid function has been used as the activation function. The proposed DL model was developed using the Keras framework (version 2.2.4) running on TensorFlow 2.0.0 in python 3.6. The hyper-parameters are summarized in Table 6.

**Table 6:** Hyper-parameters for the proposed model

Parameter	Value
Hidden layers	3
Neuron number	[17, 10, 5]
Activation functions	Sigmoid
Output activation	Sigmoid
Batch size	32
Epochs	200
Learning rate	0.001
*β*_1_	0.9
*β*_2_	0.999
ɛ	*e*^−7^

*β*_1_, Exponential decay rate of the first moment estimates; *β*_2_, Exponential decay rate of the second-moment estimates; ɛ, Small number to prevent any division by zero

### Random forest algorithm (RF)

The RF algorithm has been used as an ensemble of decision trees to make a prediction [64, 65]. A decision tree fits a function (typically piece-wise constant) over domain $X$ by recursive partitioning in a greedy way. RF regressor was used to predict the mortality risk of the patients. The following hyper-parameters have been used to train and test the RF model which is the number of estimators = 500, maximum depth = 2 and maximum feature = 5. This model was developed using the sci-kit-learn library in python.

### SMOTE technique

The Synthetic Minority Over-Sampling Technique (SMOTE) is an over-sampling approach in which the minority class is over-sampled by creating “synthetic” examples rather than by over-sampling with replacement [66]. SMOTE technique was used to balance the dataset. It generates synthetic examples in a less application-specific manner, by operating in “feature space” rather than “data space”. The minority class is over-sampled by taking each minority class sample and introducing synthetic examples along the line segments joining any/all of the K-minority class nearest neighbours [67]. In this research project, a final proportion of sm = 0.80 was set to the minority class.

1 $$sm = \frac{Majority\;class}{{Minority\;class}}$$

### Data imputation

The impact of using imputation data on the prediction model has been evaluated for cases where there are time or budget constraints and obtaining complex biomarker data is impossible or unfeasible [68, 69]. To properly evaluate the proposed imputation method, amputate available biomarker features were used to calculate the error between imputation and real values. The biomarker features were imputation for the test sets using the mean value of the “K” most similar patients from the real biomarker data and for the train set using the KNN algorithm [70]. The value of “K” is determined by the amount of available data. The benefit of using the imputation features was evaluated by comparing the model’s performance against the same test set with the real biomarker data. The error of the estimated imputation data is calculated using the root mean squared error (RMS).

2 $$RMS = \sqrt[2]{{x_{i} - \hat{x}^{2} { }}}$$

where $x$ is the real feature value and $\widehat{x}$ is the imputation feature value.

Finally, the benefit of adding imputation biomarkers data was revealed by comparing the performance of the imputation test set against the performance of a model which only uses basic patient information, without any imputation. As we impute more features, the model’s performance has more uncertainty and therefore a higher error. This motivates us to impute only the necessary number of features to see an improvement of the model without adding variance to the output.

### Training and testing data splitting

**Fig. 5:**
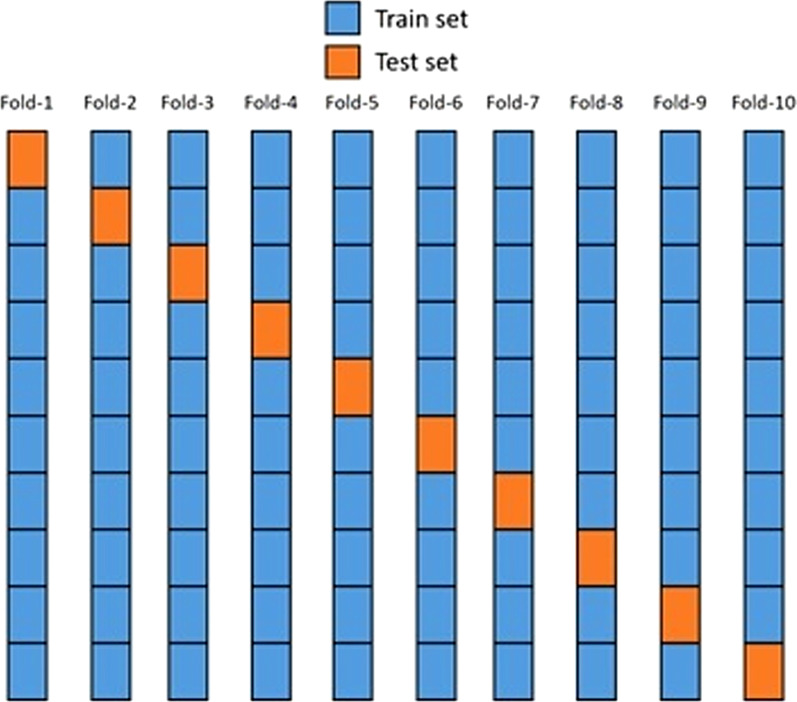
10-Fold cross-validation

To properly assess the performance of the proposed model, the datasets were divided into training and testing samples, in which 90% of the samples were for training and 10% for testing. The test samples **were** usually a small part of the dataset, only large enough to vary significantly in population. The K-fold cross-validation (CV) algorithm [71, 72] was used for every dataset. In this algorithm, the dataset was first randomly shuffled to avoid bias and then divided into K equally sized parts (folds). The proposed model was trained K times, where at each iteration a different fold, the dataset was used as the testing set while every other fold was used for training. The final unbiased result was recorded as the average value of each evaluation metric across every fold. The proportion of distribution of label classes was kept at every layer. It has only been done to avoid a fold of model training with positive or negative class patterns. For this purpose, the Stratified K-Fold sci-kit-learn function was used, which **kept** the proportion of the label feature across every fold [73–75]. Figure 5 shows a graphical representation of the 10-Fold CV algorithm.

### Decision threshold

**Fig. 6:**
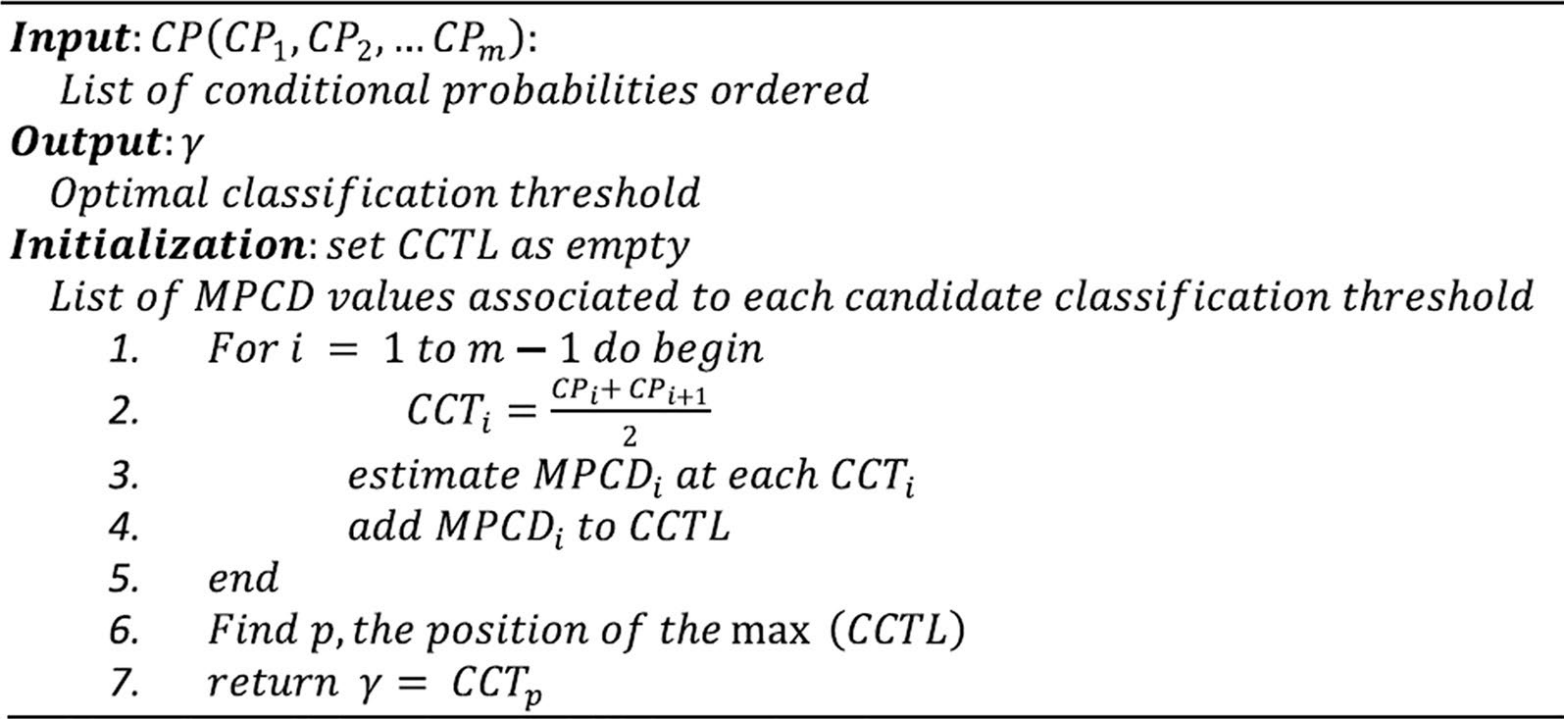
Pseudo-code of the OCTM algorithm

The decision threshold **governed** the choice to turn a forecasted probability or scores into a class label. The Optimal Classifying Threshold Method (OCTM) [76] algorithm was used to obtain the decision threshold value that optimizes the MPCD score. A 0.5 spaced decision threshold was taken for every class. The algorithm is shown in Fig. 6.

## Results and discussion

### Key performance indicators (KPIs)

To validate the proposed methods, we have used different performance indicators such as confusion matrix and its true positive (TP), false positive (FP), true negative (TN) and false-negative (FN), precision (P), sensitivity/recall (R), area under the curve (AUC), F-measure, accuracy, alpha, beta, and maximum probability of correct decision (MPCD) [77–79].

Precision (P) can be defined as the number of true positives (TP) divided by the number of TP plus the number of false-positive (FP). The P can be written as

3 $$P = \frac{TP}{{FP + TP}}$$

Recall (R) (Sensitivity) can be calculated as the number of true positives (TP) divided by the sum of the number of TP and the number of false-negative (FN). It can also be defined as the percentage of total relevant results correctly classified. The R can be posed as

4 $$R = \frac{TP}{{FN + TP}}$$

Accuracy (ACC) is referring to predicting the perfection of a machine learning model. The accuracy can be calculated as

5 $$ACC = \frac{{{\text{TN}} + {\text{TP}}}}{{{\text{FN}} + {\text{FP}} + {\text{TN}} + {\text{TP}}}}$$

Maximum Probability of Correct Decision (MPCD) is a probabilistic-based measure of classification performance aimed at analysing highly imbalanced data structures. The MPCD can be designed as

6 $${\text{MPCD}} = \left( {1 - {\text{alpha}}} \right)\left( {1 - {\text{beta}}} \right)$$

where $$alpha = \frac{FP}{{FP + TN}}$$ and $$beta = \frac{FN }{{FN + TP}}$$.

## Results

**Fig. 7:**
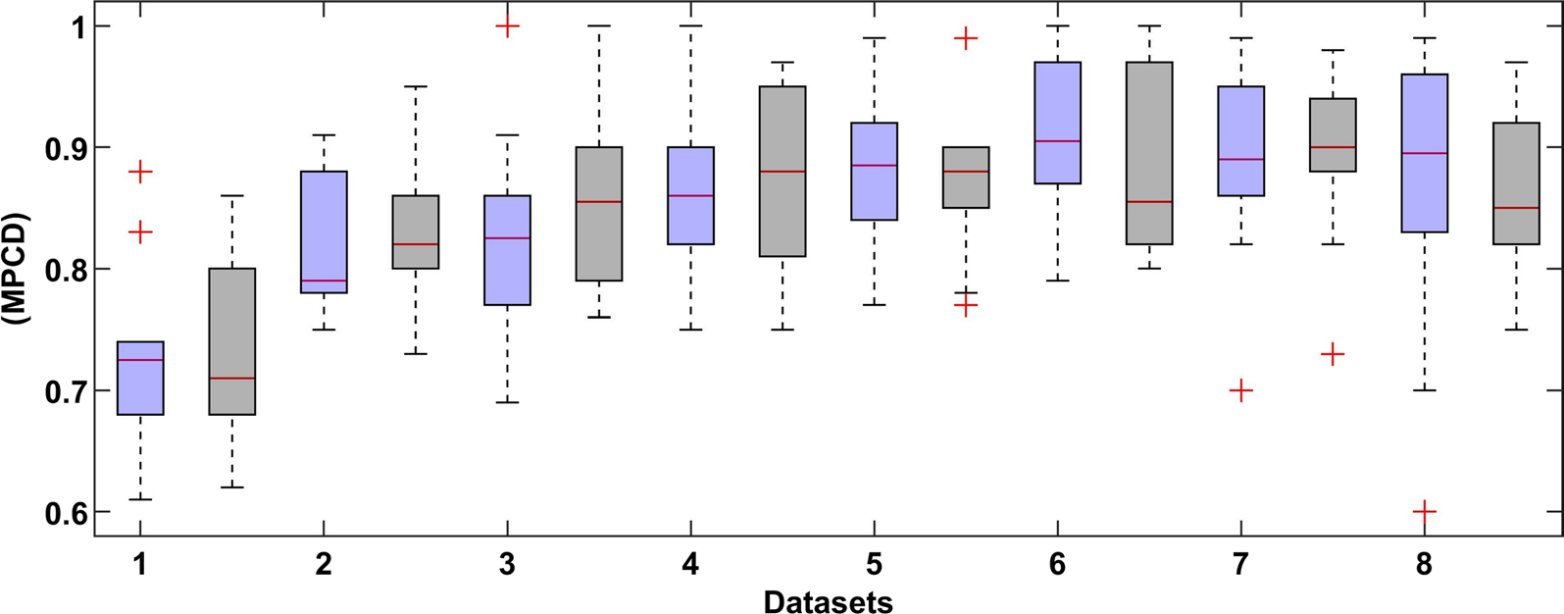
Results summary of the DL model of every dataset (the left blue colour indicates normal dataset and right black colour indicates SMOTE dataset)

**Fig. 8:**
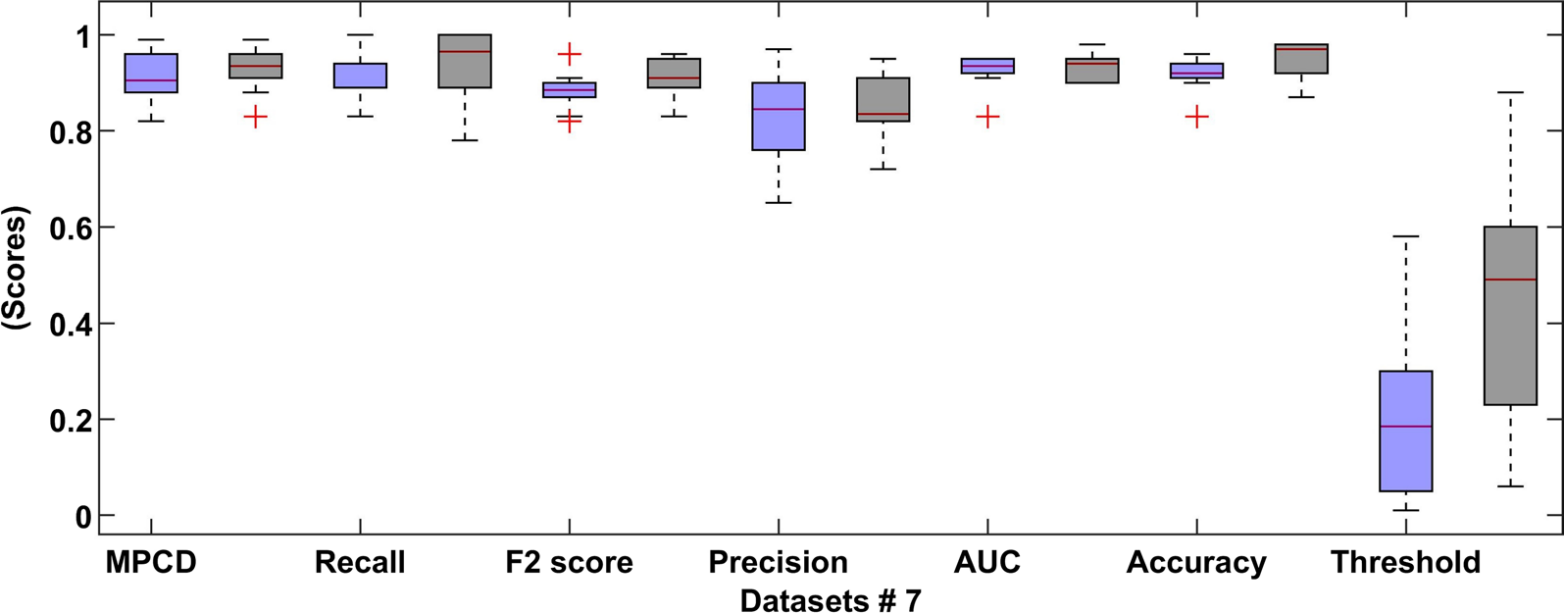
Boxplot of the tenfold CV of dataset 7 (the left blue colour indicates normal dataset and right black colour indicates SMOTE dataset)

Figure 7 shows a boxplot graph of the MPCD score of the proposed DL model with and without the SMOTE approach of every sub-dataset. The best results of the deep learning method are shown in Fig. 8.

**Fig. 9:**
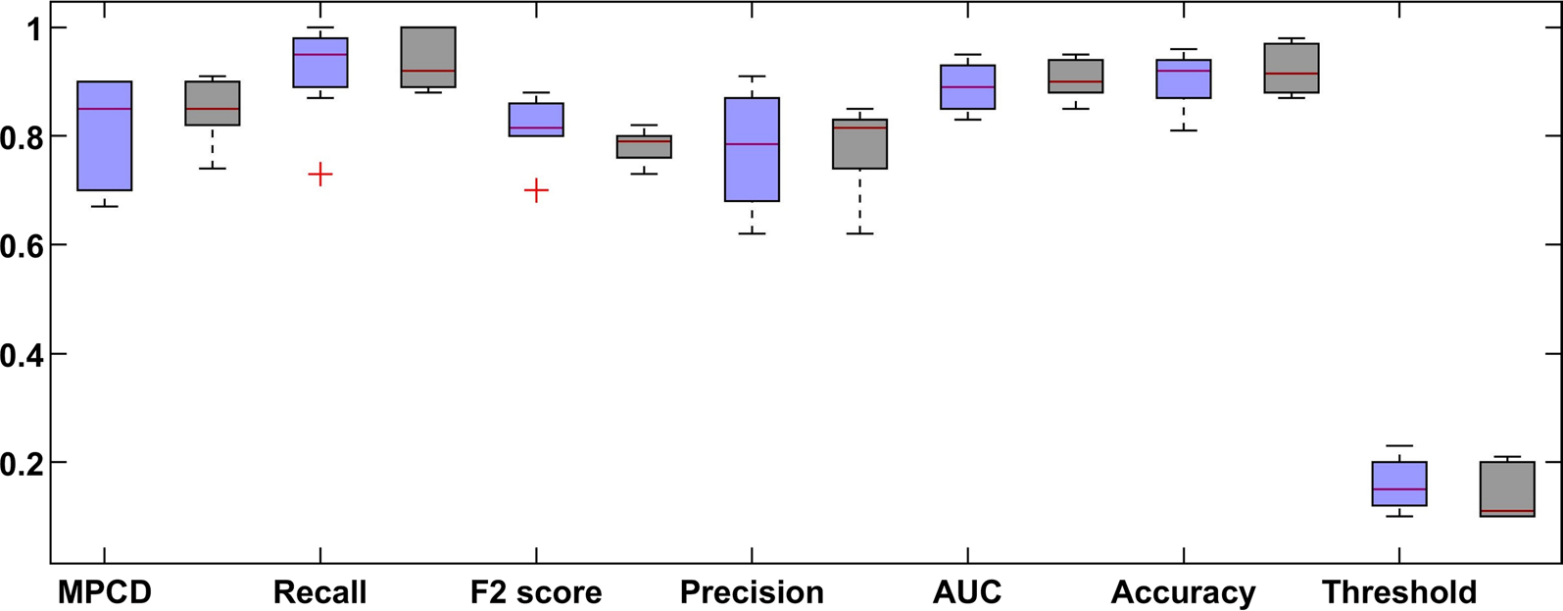
Metrics comparison of the imputation NEU%m and LIN%M biomarkers (Left-blue) against real values (Right-black)

**Fig. 10:**
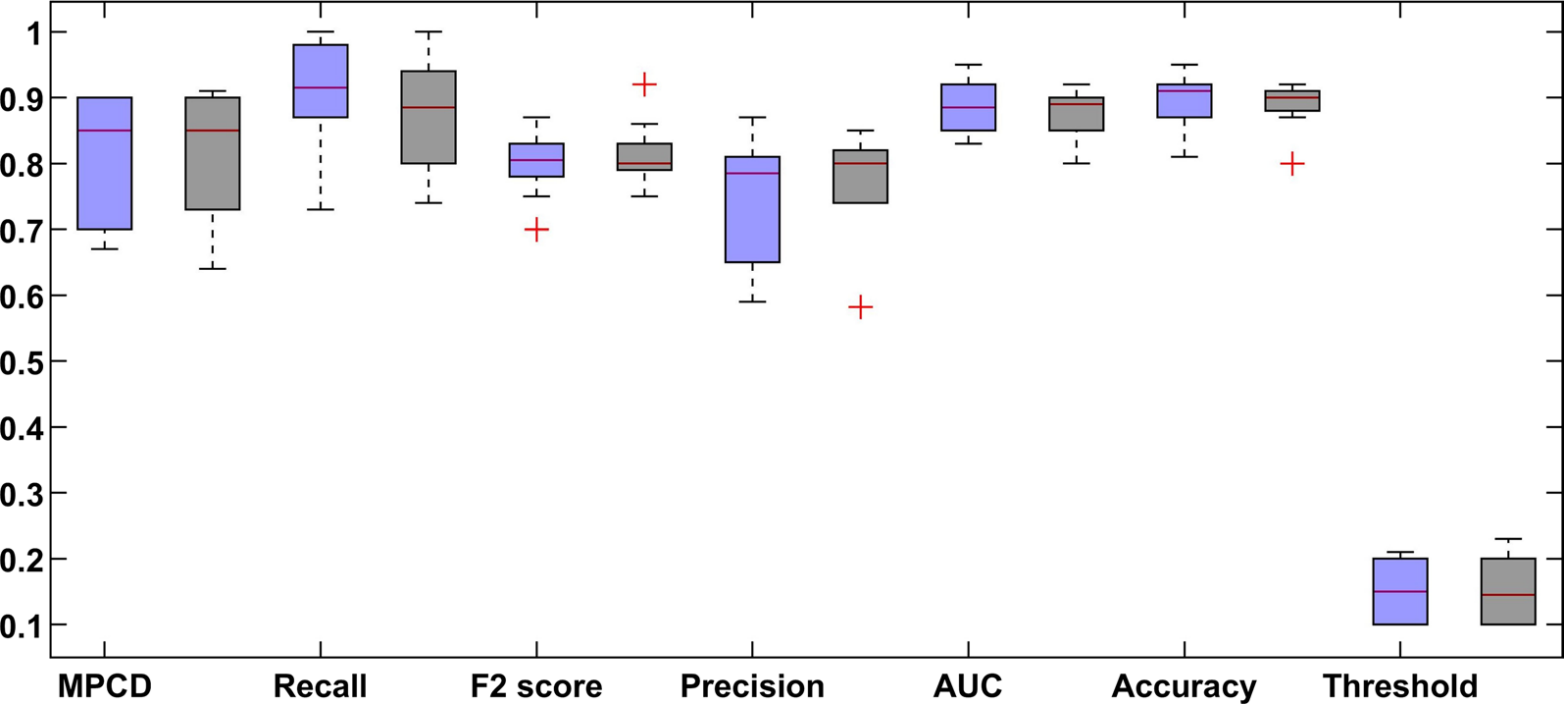
Metrics comparison of the imputation of the DDm and DDM biomarkers

Data imputation is the process of replacing missing data with substituted values. The results of the imputation scheme of the NEU%m and LIN%M biomarkers are shown in Fig. 9. The boxplot graph of every performance metric was compared with and without imputation. We can see the increase in the variance of each evaluation metric, which is expected because of the error introduced by the imputation process. The mean performance values of the model are the same overall and indicating that the imputation process did not introduce false information in the process. The results of the DDm and DDM imputation process are shown in Fig. 10. In this case, we can see a similar variance when comparing the imputation and real value models, suggesting a good imputation performance.

**Fig. 11:**
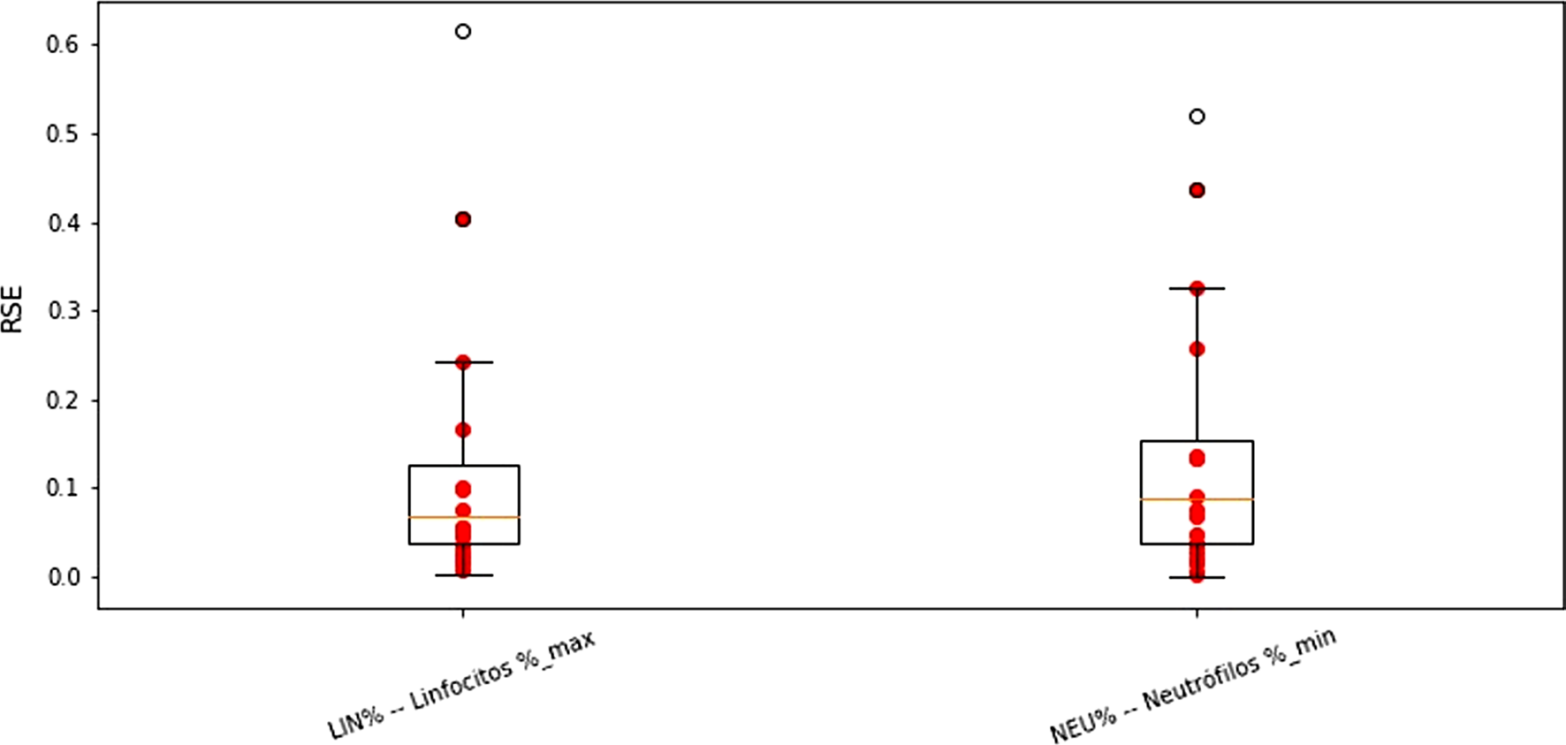
RSE values for imputation NEU%m and LIN%M features

**Fig. 12:**
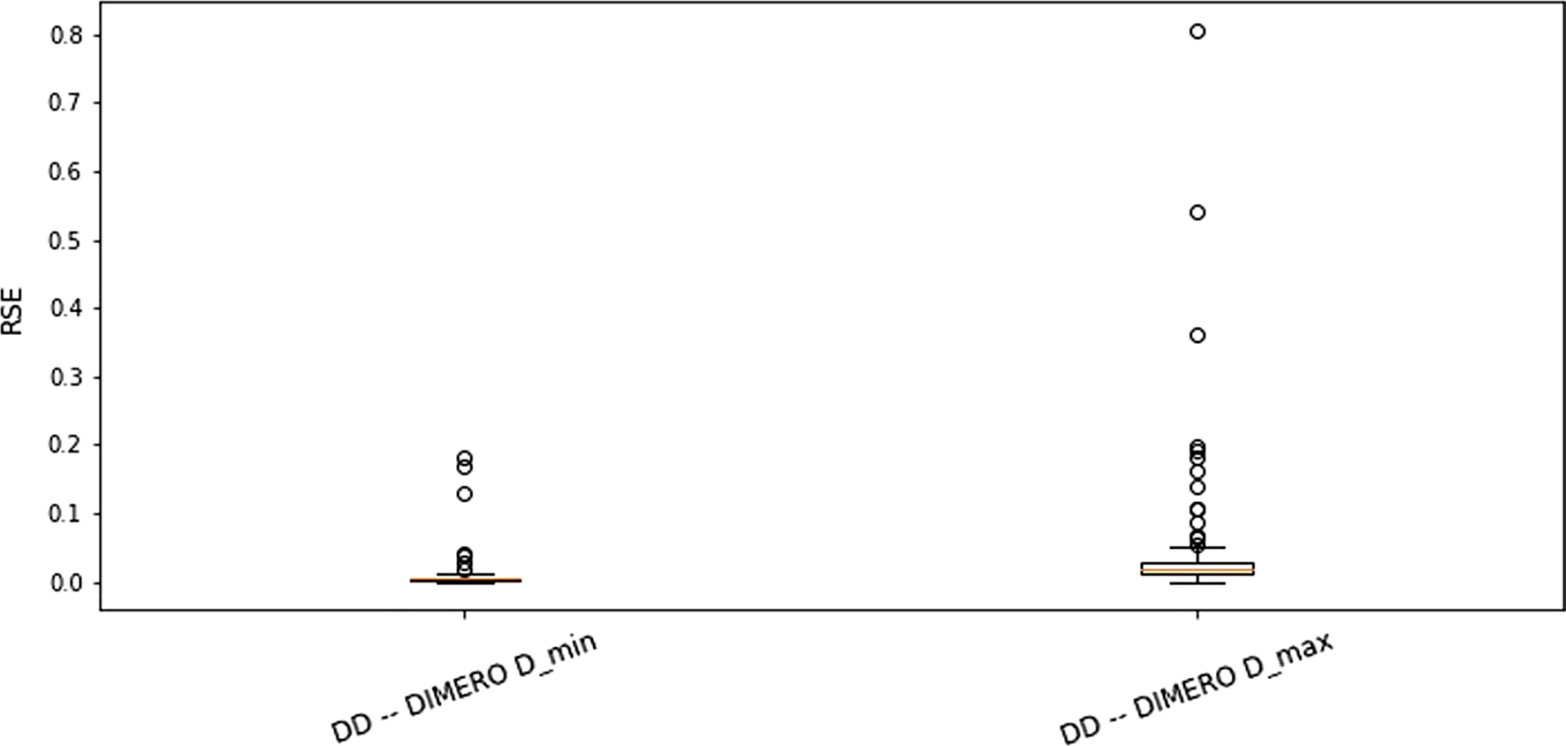
RSE values for imputation DDm and DDM features

Figures 11 and 12 show the boxplot graph of root square error (RSE) values of the 10-folds when imputation the NEU%m and LIN%M biomarkers, and DDm and DDM, respectively. The prominent red dots in the graph represent patients who had different classifications between real and imputation data.

### Performance comparison

**Fig. 13:**
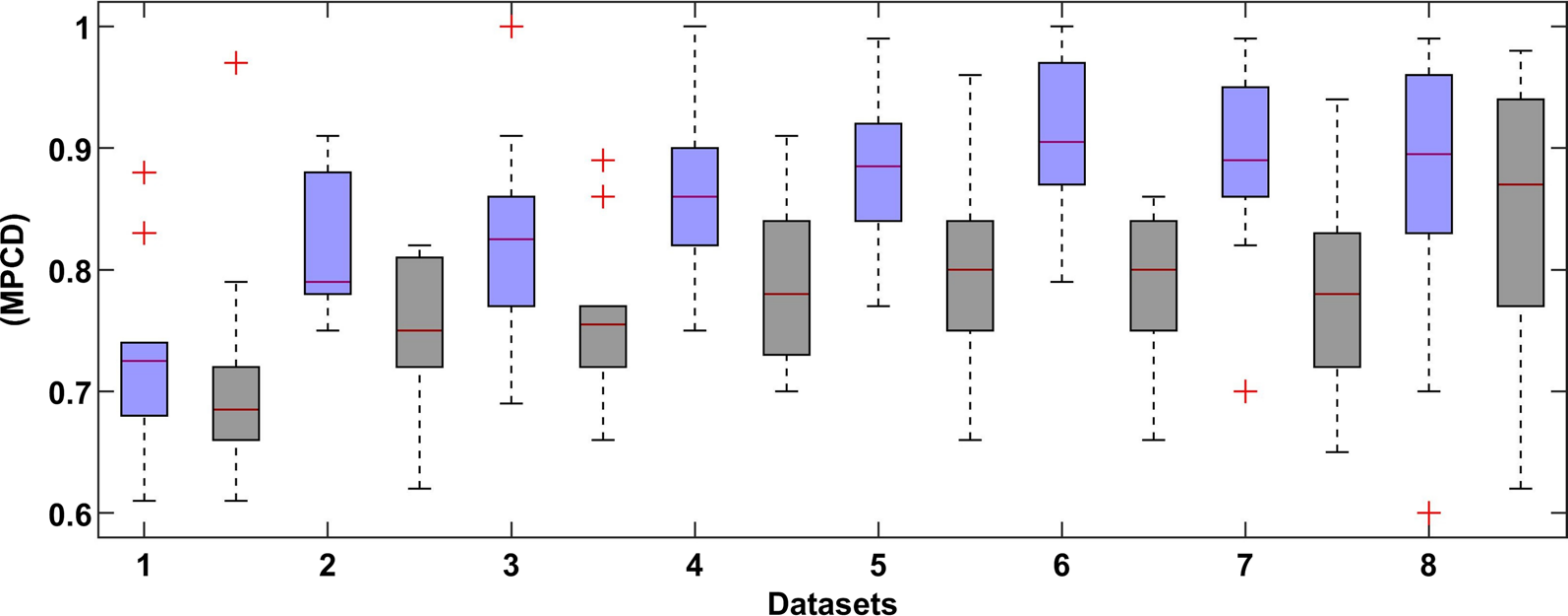
The comparative results of the DL (left-blue) and RF (right-black) models (Normal dataset)

**Fig. 14:**
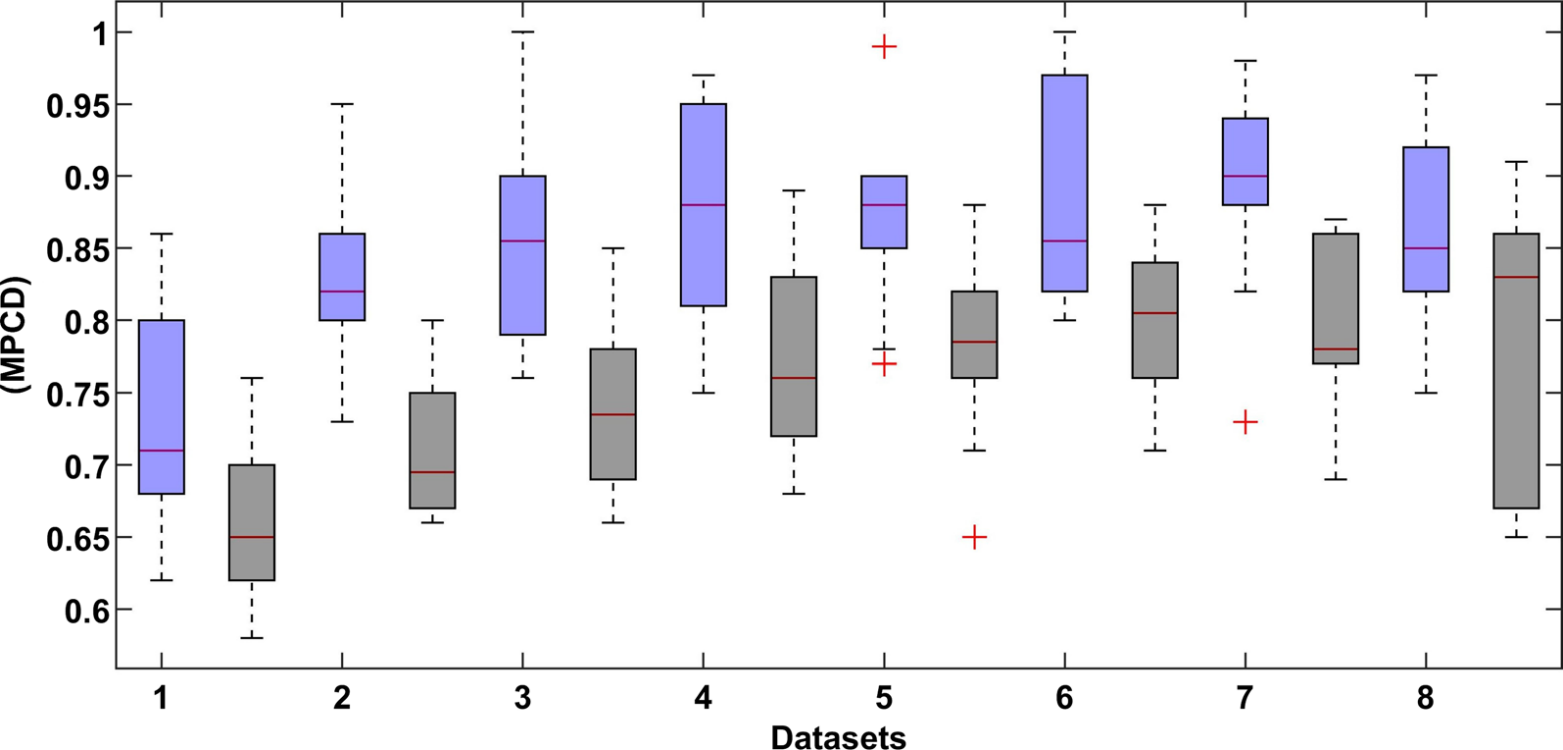
The comparative results of the DL (left-blue) and RF (right-block) models (SMOTE dataset)

For comparison purposes, the RF [64, 65] support vector machine (SVM) [80], artificial neural network (ANN) [81], XGBoost [82], logistic regression (LR) [83] algorithms have been trained and tested based on the same datasets. The comparative results of the DL and RF models of every dataset with and without the SMOTE approaches are presented in Figs. 13 and 14, respectively. The results show that the DL model has high prediction accuracy.

In Table 7, the proposed DL model outperforms comparatively the support vector machine (SVM), artificial neural network (ANN), XGBoost, logistic regression (LR) and RF model in practically every dataset on both approaches. The RF model greatly benefits from the SMOTE approach, while the DL model appears to work better even when having unbalanced classes.

**Table 7:** The proposed model (DL) is compared with other models

Algorithms	Key performance indicators (KPI) algorithm					
	MPCD	Recall	F2 score	Precision	AUC	Accuracy
DL	0.93	1	0.93	0.91	0.93	0.95
RF	0.88	0.95	0.89	0.9	0.89	0.93
SVM	0.77	0.91	0.87	0.85	0.87	0.89
ANN	0.78	0.89	0.9	0.87	0.88	0.90
XGBoost	0.81	0.93	0.94	0.90	0.90	0.91
LR	0.76	0.90	0.87	0.88	0.86	0.89

The proposed model is also compared with the recently published methods. The comparative results are shown in Table 8. In [21], the authors used 53 confirmed COVID-19 patients and the dataset was collected from the Wenzhou Central Hospital and Cangnan People’s Hospital in Wenzhou, China. The median age was 43 years, and 62.2% of patients were men. Common symptoms included fever (in 47 patients, 88.7%) and cough (in 32, 60.4%). The median number of white blood cell count (× 109 = L) was reported as 4.8, and the median number of Lymphocyte count (× 109 = L) was 1.2. Out of the 53 patients, 9.4% developed ARDS, 1.9% patients were taken into the intensive care unit (ICU) and 47.5% required supplemental oxygen. They compared various ML algorithms using a tenfold cross-validation accuracy. The top accuracy achieved was 80% using the support vector machine (SVM) and K-nearest neighbour (KNN) (k = 5) algorithm.

**Table 8:** Comparison of the proposed scheme with recently published ML models to predict COVID-19 patients' mortality risk

Algorithm	Input features dataset	Key performance indicators (KPI)					
		MPCD	Recall/sensitivity	F2 score	Precision	AUC	Accuracy
DL	48 Clinical data	0.93	1	0.93	0.91	0.93	0.95
RF		0.88	0.95	0.89	0.85	0.89	0.93
SVM		0.77	0.91	0.87	0.87	0.87	0.89
ANN		0.78	0.89	0.9		0.88	0.90
XGBoost		0.81	0.93	0.94	0.90	0.90	0.91
LR		0.76	0.90	0.87	0.88	0.86	0.89
SVM and KNN [21]	11 Clinical data	–	–	–	–	–	0.80
ANN [24]	42 Clinical data	–	–	–	–	–	0.90
ML [27]	12 Clinical data	–	0.90	–	–	0.866	–
DNN [28]	51 Clinical data	–	0.8125	–	–	0.97	0.9598
ML [29]	20 Clinical data	–	–	–	–	0.94	–
Multivariate Analysis [23] (Cox proportional regression)	4 Clinical data	–	0.95	–	–	0.91	–
ML [34]	3 Clinical data	–	–	–	–	0.91	–
Multivariate Regression model [35]	7 Clinical data	–	–	–	–	0.74	–
CNN and Deep Transfer Learning [30]	RGB X-ray images	–	0.9762	–	–	–	0.8810
CNN and Deep Transfer Learning [31]	X-ray & CT-Scan images	–	0.94	–	0.95	–	0.95
Deep CNN-LSTM [54]	X-ray Images	–	0.993	–	–	0.999	0.994
CNN- Ensemble of Machine Learning [57]	X-ray Images	–	0.978	–	1	–	0.989
CNN-RNN [53]	X-ray Images	–	0.999	–	0.999	0.999	0.999
KNN [84]	Clinical data		1.00	0.93	0.942	0.922	0.9374

Pourhomayoun et al. [24] proposed an ML algorithm to accurately predict the mortality risk of COVID-19 patients. 17,000 laboratory-confirmed COVID-19 patients’ dataset was collected from 76 countries with an average age of 56.6, from which 74.4% recovered. Data imputation techniques were used for missing values, and a balanced dataset was created for training and testing the model. 112 features were available from symptoms and doctor’s medical notes, and patient’s demographic and physiological data. After applying different filter and wrapper methods, the feature space was reduced to 42 features. The best performance accuracy (93.75%) was achieved by the ANN algorithm. Hyper-parameters were tuned using grid search and the final architecture had two hidden layers with 10 neurons in the first layer and 3 neurons in the second layer. A sigmoid function was used as the hidden layer activation function and stochastic gradient as the optimizer with a constant learning rate and a regularization rate of alpha $0.01$ was used.

In [27], taken 197 patients’ data with confirmed COVID-19 were obtained from five USA health systems including 51.3% of male patients and the majority are between 30 and 80 years old. For each patient, 12 features were extracted and fed into the model. The XGBoost classifier was shown excellent performance including the following results: sensitivity (0.90) and specificity (0.58).

In [28], the dataset consists of 10,813 patients from 52 Korean hospitals, using 51 variables for prediction. The ANN algorithm was used and got the following results accuracy = 95.98%, sensitivity = 81.25%, specificity = 96.1% and AUC = 97%.

The study comprised 2,831 patients, 711 (25.1%) of whom died during hospitalization while the remaining were discharged. Two models were trained to calculate the mortality risk using lab test results and without. The missing values were imputation using.

KNN, more than 40% of missing features were excluded, and 95% confidence intervals were calculated using bootstrapping.

The model performance evaluated using laboratory values (AUC = 93.8%) and without laboratory test values (AUC = 90.5%) [29].

Yadaw et al. [34] developed a mortality prediction model using the XGBoost algorithm. The database consisted of 3,841 patients, 8.2% deceased with features age, SpO2 and type of patient. The best results were obtained (AUC of ROC = 91%).

Ji et al. [23] used 208 patients’ dataset and the average age of 117 is 44 (56.2%), 31 (14.9%) older than 60 years, 45 (21.6%) and 40 (19.2%) patients. The clinical conditions deteriorated progressed during the observation period. Using the CALL score model, clinicians can improve the therapeutic effect and reduce mortality risk.

In [35], 641 hospitalized patients database was used with a median age of 60 years old, 40.1% female, 62% no critical illness, 30% were admitted to the ICU and 82 who expired. Five significant variables predicting.

ICU admissions were lactate dehydrogenase, procalcitonin, SpO2, smoking history, and LIN. The seven critical patients were deceased who have some other symptoms such as heart failure, procalcitonin, lactate dehydrogenase, chronic obstructive pulmonary disease, SpO2, heart rate, and old age. The mortality group uniquely contained cardiopulmonary variables. The risk score model (a multivariable regression model) yielded good accuracy with an AUC-ROC of 0.74 of the ICU admissions.

The dataset consisted of 284 X-ray images of which around 142 were positive of COVID-19. The VGG-6 image classifier was used as the top layers of the model and then added 5 layers as part of the transfer learning methodology. The proposed model achieved a sensitivity of 97.62%, specificity of 78.57%, accuracy of 88.10% and AUC-ROC of 88% [30].

In [31], proposed a CNN transfer learning model to diagnose the COVID-19 patients using X-ray and CT-scan images, and the following results were obtained including precision = 95%, recall = 94%, F1 score = 95% and accuracy = 95%.

Islam et al. [54], proposed deep CNN-LSTM algorithms for the detection of novel COVID-19 using X-ray images. The CNN algorithm was applied to extract the features and the LSTM scheme was used to detect COVID-19. The recorded KPIs include accuracy of 99.4%, AUC of 99.9%, specificity of 99.2%, the sensitivity of 99.3%, and the F1-score of 98.9%.

In [57], CNN and an ensemble of machine learning procedures were offered to detect the COVID-19 infection using X-ray images and the model performance is 98.91% accuracy, 100% precision, 97.82% recall, and 98.89% F1-score have been shown.

In [53], CNN-RNN schemes based on transfer learning were introduced to diagnose the COVID-19 infection using X-ray images and the authors also investigated four different methods using the same features. The VGG19-RNN has been judged as the best scheme with 99.9% accuracy, 99.9% AUC, 99.8% recall, and 99.8% F1- score to detect COVID-19 cases. Hence, the proposed methods are quite better for the detection of COVID-19 infection using X-ray images.

Shanbehzadeh et al. [84] evaluated different ML algorithms using 1224 hospitalized patients data with COVID-19. By comparing the performance of ML algorithms according to various evaluation criteria, the KNN algorithm with the precision of 94.21%, accuracy of 93.74%, recall of 100%, F-measure of 93.2% and ROC of 92.23%, produced better performance comparatively other algorithms.

## Discussion

In Fig. 7, the performance of the proposed model changes from dataset to dataset. This is expected because more features are used, as more information is needed to improve the behaviour of the system. However, the variability in the results **is** also increased as more samples were dropped, and more features were added, as shown in Table 5.

**Fig. 15:**
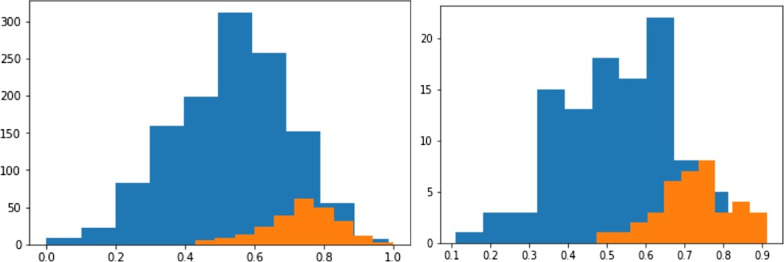
The original database class distribution on the left side, while the right side shows the DL predicted distribution

Figure 15 shows the original database class distribution on the left side, while the right side shows the DL predicted distribution. This indicates that the proposed model successfully models the dataset underlying distribution. The proposed DL model is capable of making an accurate prediction even on the unbalanced dataset. Further, analysing the proposed DL outcomes distributions is very close to the actual output distribution of the dataset.

In Fig. 8, the comparative results show that the SMOTE approach has a recall distribution that is closer to 1, but more variability in the precision metric. This is seen on the outlay of MPCD value around 0.95 which may imply a possible improvement in the model performance while introducing more training data. The same can be said about the final dataset, where the variability of the MPCD score is bigger, but outliers with very high MPCD values are also observed. Finally, the threshold value set by the SMOTE approach gets closer to 0.50 because the proportion between classes is close to one another. We observed a large variation in each evaluation metric, which is expected because of the high imbalance dataset.

The recall metric was further analyzed to quantify how good the predictions are. As we know the recall metric shows the proportion of the positive samples correctly classified. In Figs. 8, 9 and 10, the distribution of the recall metric of the dataset, the mean recall value is 0.92, which means that 92% confidence of correctly classifying any positive prediction. Additionally, we can see 10-folds where the recall value reaches 1.00, indicating that no positive samples were misclassified.

Figure 11 shows the boxplot graph of root square error (RSE) values of the 10-folds when imputation the NEU%m and LIN%M biomarkers, it can be seen more variance in the imputation values and have more error. Also, it can be observed that most of the patients classified differently when using imputation are above the 3^rd^ quartile for the observed error distribution. This can recommend a lack of information in the current database to properly imputation these features.

Figure 12 shows that the overall RSE when imputation the DDm and DDM features are smaller, with very low variance and just a couple of outliers. Imputation of these features should yield a very similar result to the real feature value.

However, comparative analysis shows that the proposed DL method yields substantially higher results for clinical and biomarker datasets. The proposed DL model can make an accurate prediction even on the unbalanced dataset. The proposed procedure can be applied to research areas such as manufacturing.

## Conclusion and future work

There is still not much we understand about the COVID-19 disease and its high reproduction rate calls on hospitals to predict the evolution of the patient on admission to effectively manage hospital resources. A mortality risk calculator for COVID-19 patients is proposed based on the DL model, and the five different algorithms have been tested including RF, SVM, ANN, XGBoost, and LR to calculate the risk of mortality of patients with COVID-19 infection using the same features and datasets. Therefore, a mortality risk calculator must not only accurately classify patients with high mortality risk, but it is also working on the necessary features. This can enable hospitals to make early predictions even when only basic features are available while evaluating the benefits of later obtaining more complex biomarker features. The proposed DL model was tested using only the most basic features had an average MPCD score of 0.75, while the best MPCD score was 0.86 obtained using 24 input features, 16 basic and 8 biomarker data (both the maximum and minimum values).

The proposed model DL shows significantly excellent results when evaluating each of the proposed datasets. Both over-sampling and data imputation approaches were analysed. The data imputation method based on the KNN algorithm was proposed and employed to improve the MPCD results. The proposed imputation strategy improved the MPCD (0.75) and recall (0.92) scores while only imputation 2 features. In addition, to predict the risk of death, falsely if a patient has a lower risk of death, it is far more critical than the other way around. Therefore, false negatives should be prioritized over false-positive predictions.

Both imputation results indicate that the model’s performance can indeed benefit from the imputation of said biomarkers. The recall metric got an overall mean value of around 0.90 which outperforms the 0.87 of the models without any imputations, while also reaching recall values of about 0.95.

The analysis presented in this research project can be applied to other research areas, e.g., finance or manufacturing. In the defect detection or prediction problem in the manufacturing area, where the positive (defect) to negative (non-defective) ratio is also very unbalanced, the prediction problem can be analysed similarly.

Future work: Evaluate the effect of data imputation for complex biomarker data. Add other types of statistical representation for biomarkers time series data, by standardizing sampling frequency of both vital signs and lab test results. Test usage of a time series dedicated algorithm, i.e., Recurrent Neural Networks, ARMA models, etc. to predict patient’s evolution through time. Evaluate data imputation efficiency for every biomarker feature in a greedy way.

## Data Availability

The data presented in this study are available on request from the corresponding author.
